# Differential Regulation of Interferon Signaling Pathways in CD4^+^ T Cells of the Low Type-2 Obesity-Associated Asthma Phenotype

**DOI:** 10.3390/ijms221810144

**Published:** 2021-09-20

**Authors:** Fahd Alhamdan, Leigh M. Marsh, Frauke Pedersen, Bilal Alashkar Alhamwe, Clemens Thölken, Petra Ina Pfefferle, Thomas Bahmer, Timm Greulich, Daniel P. Potaczek, Holger Garn

**Affiliations:** 1 Translational Inflammation Research Division & Core Facility for Single Cell Multiomics, Member of the German Center for Lung Research (DZL) and the Universities of Giessen and Marburg Lung Center, Medical Faculty, Philipps University of Marburg, D-35043 Marburg, Germany; alhamdaf@staff.uni-marburg.de (F.A.); danppot@gmail.com (D.P.P.); 2 Ludwig Boltzmann Institute for Lung Vascular Research, A-8010 Graz, Austria; leigh.marsh@lvr.lbg.ac.at; 3 Lungen Clinic Grosshansdorf GmbH, Airway Research Center North (ARCN), Member of the German Center for Lung Research (DZL), D-22927 Großhansdorf, Germany; f.pedersen@pulmoresearch.de (F.P.); t.bahmer@lungenclinic.de (T.B.); 4 Clinic for Hematology, Oncology and Immunology, Center for Tumor Biology and Immunology, Institute of Tumor Immunology, Medical Faculty, Philipps University of Marburg, D-35043 Marburg, Germany; bilal.alashkaralhamwe@staff.uni-marburg.de; 5 College of Pharmacy, International University for Science and Technology (IUST), Daraa 15, Syria; 6 Institute of Medical Bioinformatics and Biostatistics, Medical Faculty, Philipps University of Marburg, D-35037 Marburg, Germany; thoelken@uni-marburg.de; 7 Comprehensive Biobank Marburg (CBBMR), Member of the German Biobank Alliance (GBA) and the German Center for Lung Research (DZL), Medical Faculty, Philipps University of Marburg, D-35043 Marburg, Germany; pfefferl@med.uni-marburg.de; 8 Department for Internal Medicine I, Campus Kiel, Airway Research Center North (ARCN), Member of the German Center for Lung Research (DZL), University Hospital Schleswig-Holstein, D-24105 Kiel, Germany; 9 Pulmonary and Critical Care Medicine, Member of the German Center for Lung Research, University Medical Center Giessen and Marburg, Department of Medicine, D-35043 Marburg, Germany; greulich@med.uni-marburg.de

**Keywords:** low type-2 asthma, obesity, asthma phenotypes, CD4^+^ T cells, transcriptomics

## Abstract

In the era of personalized medicine, insights into the molecular mechanisms that differentially contribute to disease phenotypes, such as asthma phenotypes including obesity-associated asthma, are urgently needed. Peripheral blood was drawn from 10 obese, non-atopic asthmatic adults with a high body mass index (BMI; 36.67 ± 6.90); 10 non-obese, non-atopic asthmatic adults with normal BMI (23.88 ± 2.73); and 10 healthy controls with normal BMI (23.62 ± 3.74). All asthmatic patients were considered to represent a low type-2 asthma phenotype according to selective clinical parameters. RNA sequencing (RNA-Seq) was conducted on peripheral blood CD4^+^ T cells. Thousands of differentially expressed genes were identified in both asthma groups compared with heathy controls. The expression of interferon (IFN)-stimulated genes associated with IFN-related signaling pathways was specifically affected in obese asthmatics, while the gap junction and G protein-coupled receptor (GPCR) ligand binding pathways were enriched in both asthma groups. Furthermore, obesity gene markers were also upregulated in CD4^+^ T cells from obese asthmatics compared with the two other groups. Additionally, the enriched genes of the three abovementioned pathways showed a unique correlation pattern with various laboratory and clinical parameters. The specific activation of IFN-related signaling and viral infection pathways might provide a novel view of the molecular mechanisms associated with the development of the low type-2 obesity-associated asthma phenotype, which is a step ahead in the development of new stratified therapeutic approaches.

## 1. Introduction

Over the last decades, non-communicable diseases (NCDs) have become the major cause of death worldwide, especially after the development of effective anti-infectious measures of prevention (vaccines) and treatment (antibiotics) [1,2,3]. Moreover, they represent a huge burden on the healthcare system and economic situation [4,5]. Prevalence and incidence of NCDs are still further increasing along with ongoing urbanization, industrialization, and globalization of unhealthy diet and lifestyles [6,7]. According to the World Health Organization (WHO), the rapid increase in the NCDs is mainly driven by various risk factors including tobacco, harmful use of alcohol, and obesity [8]. Furthermore, different NCDs and their major risk factors seem to have substantially overlapping underlying mechanisms, often involving immunometabolic alterations [9,10]. Combinations of certain NCDs and associated risk factors can create separate specific disease entities, classified as a clinical subtype or phenotype [11].

Obesity is a key risk factor underlying a variety of major NCDs, including asthma [10,12]. The comorbidity of obesity and asthma is referred to as obesity-associated asthma [13,14]. Various associated clinical characteristics of obesity and asthma have been described. The major obesity-associated asthma phenotype is characterized as “late-onset, severe and difficult to treat, type-2 low inflammation disease and presenting mostly in women” [15,16,17]. While these detailed clinical characteristics strictly define this particular phenotype of asthma, the specific underlying cellular and molecular mechanisms of this phenotype are still only poorly understood.

Gene expression alterations, especially in cells critically involved in disease development, represent a major molecular contributor to the pathophysiology of chronic inflammatory diseases such as asthma. Subsets of CD4^+^ T cells, such as Th1, Th2, and Th17 have been shown to differentially contribute to the initiation and perpetuation of specific asthma phenotypes [18,19]. Certain biological pathways and cellular processes play an essential role in the polarization of the CD4^+^ T cells subtypes [20]. Thus, deep understanding of how those molecular mechanisms underlie clinical asthma representations might help to further solve the complex disease heterogeneity puzzle, particularly also with regard to specific aspects of obesity-associated asthma phenotypes.

The current study addresses the specific role of CD4^+^ T cells in obesity-associated low type-2 asthma. We compared the transcriptome profiles of these cells in strictly selected, clinically characterized obese and non-obese low type-2 asthmatics and related the findings to healthy controls. In addition, functional pathway enrichment and gene clusters network analyses were conducted to comprehensively understand the association of the dysregulated genes with the respective asthma phenotype.

## 2. Results

### 2.1. Patient Selection and Clinical Parameters Comparison

In the present study we selectively focused on low type-2 asthmatics as identified by low blood eosinophil counts (<300 eos/µL) and low FeNO (<25 ppb) levels according to the latest Global Initiative for Asthma (GINA) [21], associated with low serum total IgE levels (<300 kU/L). A detailed comparison of the two asthma groups based on laboratory and clinical parameters including CRP, FeNO, age of onset, and lung function parameters are provided in Table 1. With the exception of BMI, no significant differences in clinical parameters were identified between the obese asthmatics and non-obese asthmatics groups. 

To confirm the low type-2 state of the asthmatic patients, we checked the expression of a series of recently identified marker genes of Th2 differentiation [22] in the CD4^+^ T cells transcriptome data set. The majority of the tested Th2 genes showed no significant differences between either asthma group when compared with healthy controls (Table 2). Interestingly, the master transcription factor for the differentiation and activation of the Th2 cell subset, i.e., GATA binding protein 3 (*GATA3*), was significantly less expressed in both asthma groups compared with healthy controls, while expression of interleukin 4 (*IL-4*), *IL-5*, and *IL-13* were below the detection limit for all samples.

### 2.2. Differentially Expressed Genes (DEGs) of CD4^+^ T Cells in Low Type-2 Obese and Non-Obese Asthmatics

**Figure 1:**
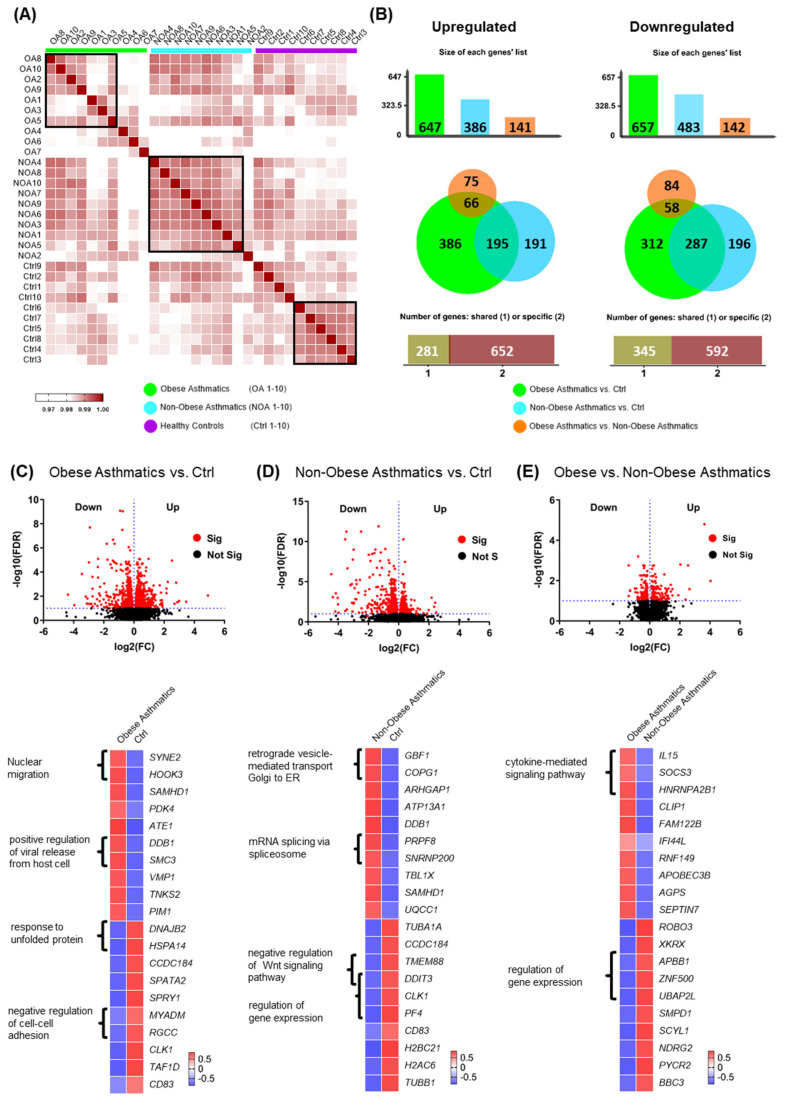
Differentially expressed genes of both low type-2 obese asthma and non-obese asthma phenotypes using a significance threshold cutoff for FDR < 0.1. (**A**) Correlation matrix of 75% of the detected genes for 10 obese asthmatics ”OA, green”, 10 non-obese asthmatics “NOA, turquoise”, and 10 healthy controls “Ctrl, purple”. (**B**) Proportional Venn diagrams showing the specific and shared genes of obese asthmatics vs. Ctrl (healthy controls), non-obese asthmatics vs. Ctrl, and obese asthmatics vs. non-obese asthmatics. (**C**–**E**, up) Volcano plots presenting differentially expressed genes of the three comparisons. (**C**–**E**, down) Heatmaps showing the top significantly 10 up and downregulated genes with some of the associated biological processes (GO terms, *p* < 0.05).

To identify specific transcriptome patterns in CD4^+^ T cells associated with low type-2 obese asthma, we performed differential gene expression analyses in peripheral blood-derived CD4^+^ T cells of 10 obese asthmatic adults in comparison with 10 non-obese asthmatics as well as 10 healthy non-obese adults. Following RNA sequencing (RNA-Seq) and bioinformatic analysis, we first created a correlation matrix based on the 75% most abundantly expressed genes in order to observe distance and clustering of the matched samples (Figure 1A). All three study groups exhibited major unique clusters and some smaller sub-clusters. Interestingly, the obese asthmatics showed less clustering consistency than non-obese asthmatics. Further, we performed a pairwise comparison of the three studied groups at a significance threshold for the false discovery rate (FDR) < 0.1. A total of 1304 DEGs (647 up and 657 down) were identified in CD4^+^ T cells of obese asthmatics compared with healthy controls (Ctrl), while 869 genes (386 up and 483 down) were found to be differentially expressed in CD4^+^ T cells of non-obese asthmatics compared with healthy controls. A total of 283 DEGs (141 up and 142 down) were detected when comparing CD4^+^ T cells from obese asthmatics with those from non-obese asthmatics (Figure 1B). As shown in the Venn diagram in Figure 1B, 386 genes were found to be specifically upregulated in the comparison of obese asthmatics vs. Ctrl (green circle), while 195 and 66 upregulated genes were shared between non-obese asthmatics vs. Ctrl and obese asthmatics vs. non- obese asthmatics comparisons, respectively (Figure 1B, left). Likewise, the numbers of significantly downregulated genes for all three comparisons are shown in the right panel of (Figure 1B).

To separately investigate each comparison in more details, we created volcano plots (Figure 1C–E, up) and heat maps depicting the top 10 significantly up and downregulated genes alongside their associated biological processes (GO terms; Figure 1C–E, down). 

Our results demonstrated the *positive regulation of viral release from the host cell pathway* to be enriched in the obese asthmatics vs. healthy controls as represented by the upregulated genes damage-specific DNA-binding protein 1 (*DDB1*) and structural maintenance of chromosomes 3 (*SMC3*) (Figure 1C, down). The *negative regulation of Wnt signaling pathway* was less active in non-obese asthmatics compared with healthy controls, as represented by the downregulated transmembrane protein 88 (*TMEM88*) and DNA damage-inducible transcript 3 (*DDIT3*) genes (Figure 1D, down). In the comparison of obese asthmatics vs. non-obese asthmatics, the upregulated *IL15*, *SOCS3*, and heterogeneous nuclear ribonucleoprotein A2/B1 (*HNRNPA2B1*) genes were associated with the *cytokine-mediated signaling pathway* (Figure 1E, down). Further differentially expressed genes and their related biological processes are shown in Figure 1C–E.

### 2.3. Predominant Interferon Signaling Pathway in CD4^+^ T Cells of Low Type-2 Obese Asthmatics

**Figure 2:**
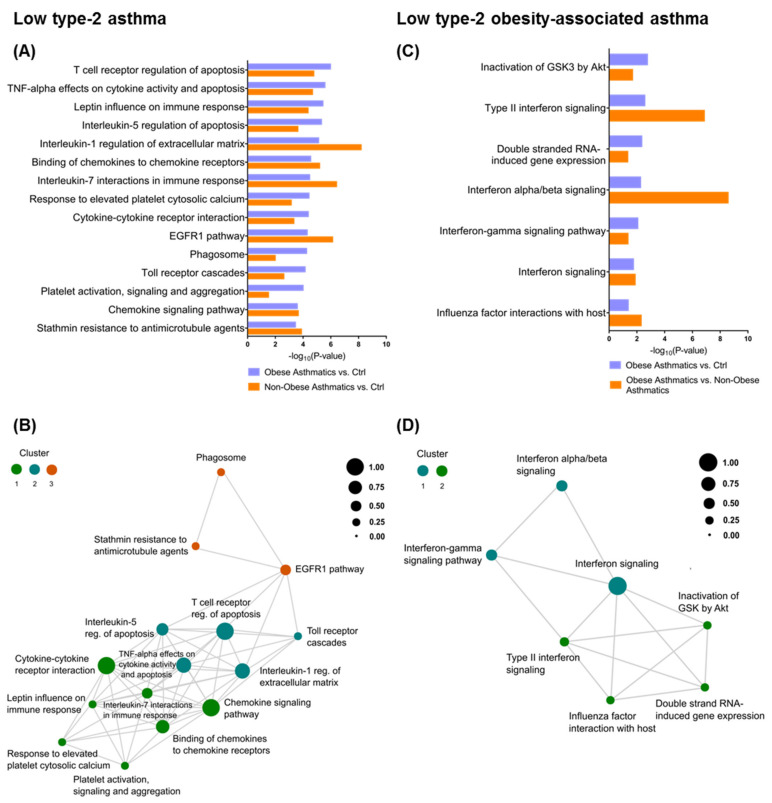
Biological pathway analysis utilizing Bioplanet database with significance threshold of *p* < 0.05. (**A**) Bar plot presenting the top 15 significant pathways shared between obese asthmatics vs. Ctrl (healthy controls) and non-obese asthmatics vs. Ctrl, alongside their (**B**) network analysis showing the association between the previous pathways (circle color corresponds to pathways cluster, circle size to the number of mutual genes between the corresponding pathways and all other connected pathways, circles proximity to the number of mutual genes between two pathways). (**C**) Bar plot presenting all significant pathways shared between asthmatics vs. Ctrl and obese asthmatics vs. non-obese asthmatics with their (**D**) network analysis.

We further carried out a functional pathway enrichment analysis utilizing the genes comprising a fold change (FC) ≥ 1.5 at FDR < 0.1. The joined pathway analysis of obese and non-obese asthmatics compared with healthy controls revealed a variety of shared biological pathways at different significance levels in the two asthma groups. The top 15 significant canonical pathways are shown in Figure 2A. To elucidate the interconnection between the enriched pathways, we identified genes mutually involved in the respective pathways and performed a correlation analysis based on these genes (Supplementary Figures S1A and S1B). Three distinct pathway clusters were identified. Further, we performed pathway network analysis based on the two previous analyses. Pathways such as *cytokine–cytokine receptor interaction and chemokine signaling pathway* encompassed the highest number of mutual genes (size of circle) in the first pathways cluster (green) and provided a scaffold to the second pathways cluster (blue) (Figure 2B). Next, we applied the same analysis to the obese asthmatics vs. Ctrl and obese asthmatics vs. non-obese asthmatics comparisons in order to highlight the obesity asthma-related pathways. Interestingly, pathways representing *interferon signaling* and *viral infection* mechanisms were robustly overrepresented in this analysis (Figure 2C). Notably, the *EIF2AK2* gene, encoding eukaryotic translation initiation factor 2 alpha kinase 2, also known as interferon-induced double-stranded RNA-activated protein kinase, coincided with most of the pathways and might thus be critical for specific pathomechanisms of the obesity-associated asthma phenotype (Supplementary Figure S1C). We detected two main clusters of pathways in the corresponding analysis, clearly demonstrating a central role for interferon signaling mechanisms (Supplementary Figures S1D and Figure 2D).

### 2.4. Further Gene Regulation Pathways Involved in Low Type-2 Asthma Phenotypes

**Figure 3:**
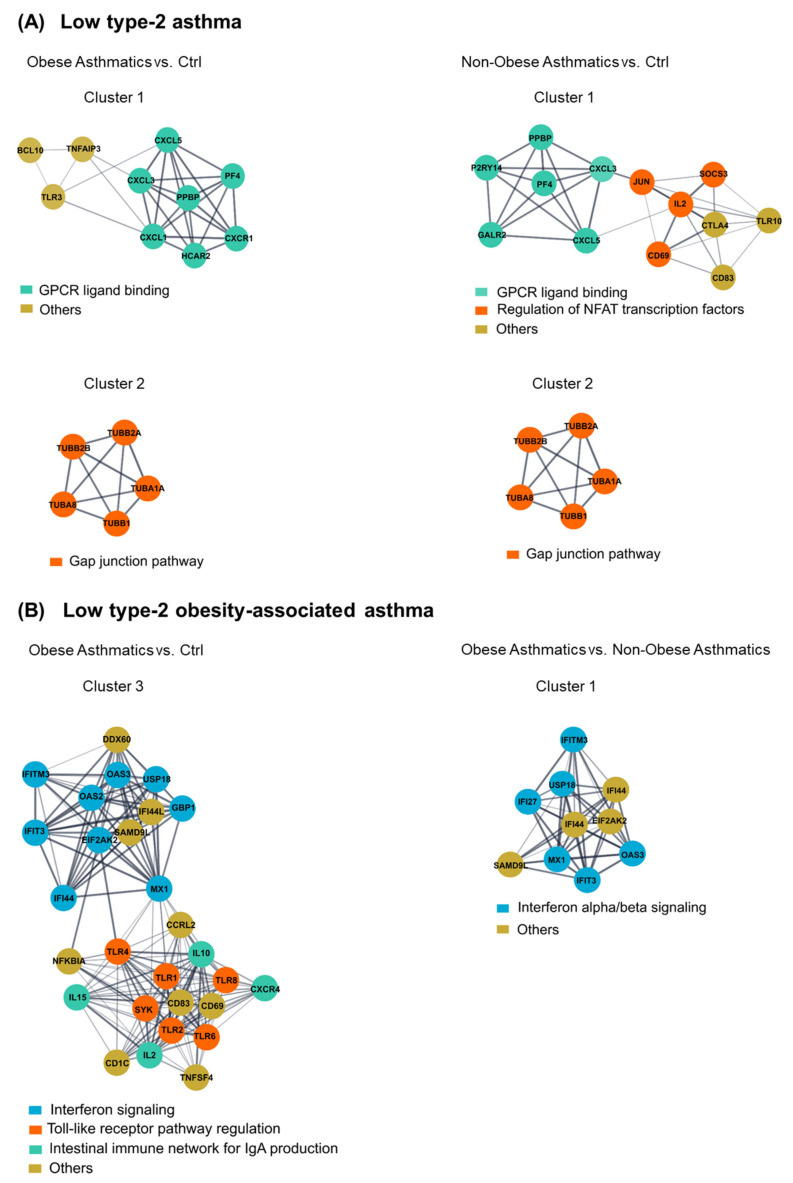
Clustering of dense network regions and their associated biological pathways. Gene clusters associated with shared pathways between ((**A**), left) obese asthmatics vs. Ctrl (healthy controls) and ((**A**), right) non-obese asthmatics vs. Ctrl, as well as between ((**B**), left) obese asthmatics vs. Ctrl and ((**B**), right) obese asthmatics vs. non-obese asthmatics.

To select candidate biological pathways from the previous analysis, we performed gene network and clustering analyses for each of the three comparisons separately and chose the clusters containing the shared pathways. These network gene clusters were built from all genes of each of the three comparisons showing a FC ≥ 1.5 at FDR < 0.1. Further, the huge STRING PPI gene networks were subdivided into clusters based on topology to find densely connected network regions. Genes of the *G protein-coupled receptor (GPCR) ligand binding* and *gap junction pathways* were identified in both comparisons of obese asthmatics vs. Ctrl and non-obese asthmatics vs. Ctrl (Figure 3A). Moreover, in the obese asthmatics vs. Ctrl and vs. non-obese asthmatics comparisons, the *interferon signaling pathway* was again featured as differentially affected, identifying this pathway as a quite specific mechanism in the regulation of CD4^+^ T cells activities of obese low type-2 asthmatics (Figure 3B). Differential expression of genes in the gene network clusters are shown in Supplementary Figure S2.

### 2.5. Correlation of Biological Pathway Enriched Genes with Clinical Variables

**Figure 4:**
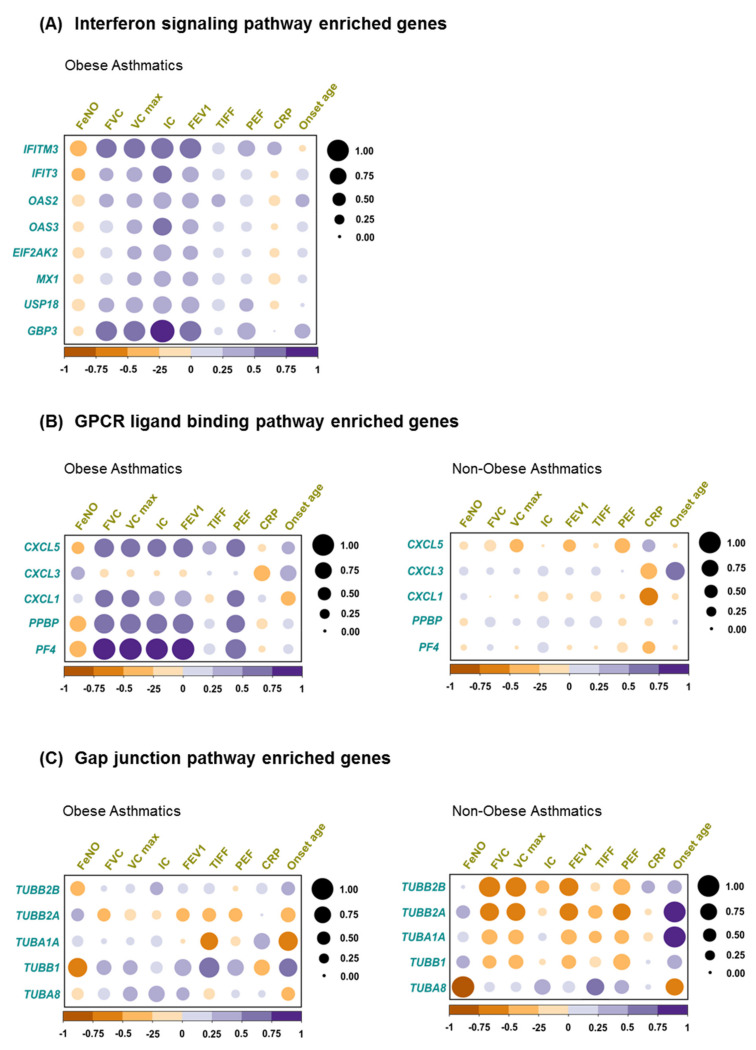
Correlation between clinical variables and biological pathways enriched genes. Pearson correlation between clinical assessments and (**A**) interferon signaling pathway enriched genes in obese asthma, (**B**) gap junction pathway enriched genes in both obese asthmatics and non-obese asthmatics, and (**C**) GPCR ligand binding enriched genes in both obese asthmatics and non-obese asthmatics.

As shown in the patients’ characteristics (Table 1), asthma-related clinical parameters revealed no significant differences between obese asthmatics and non-obese asthmatics and cannot be used to distinguish between these phenotypes. However, we intended to explore the correlation between these parameters and gene expression levels in the two asthmatics populations. Thus, we exploited the enriched genes represented in the three major canonical pathways contributing to differentially regulated network clusters, i.e., *IFN signaling*, *GPCR ligand binding*, and *gap junction pathways*. In obese asthmatics strong positive correlations of interferon-induced transmembrane protein 3 *(IFITM3*) and guanylate-binding protein 3 (*GBP3*) with peak expiratory flow (PEF), forced expiratory volume in the first second (FEV1), forced vital capacity (FVC), maximum vital capacity (VC max), and inspiratory capacity (IC) were identified, while some less pronounced inverse correlations were observed between further *IFN signaling pathway* enriched genes and FeNO (Figure 4A). 

Additionally, in obese asthmatics, except C-X-C motif chemokine ligand 3 (*CXCL3*), *GPCR binding ligand* enriched genes correlated positively with FVC, VC max, IC, FEV1, and PEF (Figure 4B, left), while this correlation pattern was not observed in non-obese asthmatics (Figure 4B, right). This might indicate the presence of a superior clinical-molecular link specifically in the obese asthmatics.

Eventually, in case of *gap junction pathway* enriched genes, tubulin alpha 1a (*TUBA1A*) gene showed a robust negative correlation with TIFF and onset age, whereas tubulin beta 1 class VI (*TUBB1*) gene correlated negatively with FeNO in obese asthmatics (Figure 4C, left). In contrast, in non-obese asthmatics, most of the gap junction pathway enriched genes exhibited a negative correlation with PEF, FEV1, FVC, and VC max (Figure 4C, right).

## 3. Discussion

Even though our understanding of asthma has substantially increased within the last several decades, the progress made is not sufficient to fully understand the molecular mechanisms behind various clinical phenotypes of the disease, including obesity-associated asthma, i.e., to identify the relevant underlying molecular endotypes [23,24]. In the age of personalized medicine, this may represent an important basis for the establishment of highly detailed methods of molecular endotyping. This would enable the identification of asthmatics benefiting the most from specific treatment modalities or the selection of stratified therapeutic options for specific patients [25,26]. Therefore, we here performed a gene expression analysis in patients with the low type-2 obesity-associated asthma phenotype. To perform this task in a comprehensive and systematic way, we (i) conducted mRNA analyses on a genome-wide level, (ii) applied a stepwise bioinformatics analysis from DEGs through enriched pathways to select the pathways with the highest biological relevance, (iii) compared results with low type-2 asthmatics with no accompanying obesity and included a healthy control group, and (iv) performed all the analyses in a highly purified population of CD4^+^ T cells. These cells are known to play a central regulatory role in asthma development by orchestrating the actions of a variety of effector cells [27,28,29]. Thus, understanding their differential biology based on gene expression patterns is a major step forward in understanding the pathophysiology of asthma, and more specifically of the obesity-related asthma phenotype.

Although standard laboratory and clinical measurements such as lung function parameters, CRP, FeNO, and age of onset were not able to distinguish between the obese and non-obese asthma groups, there was an obvious difference between those two groups by hundreds of differentially expressed genes in CD4^+^ T cells. Generally, we observed a greater variability in gene expression of CD4^+^ T cells of obese asthmatics compared with non-obese asthmatics. This might be due to varying stimulating signals triggered by adipose tissue or other potential factors that exert immunomodulatory effects (Figure 1A) [30,31]. Among the top 10 significant DEGs, *IL15* and *SOCS3* were found to be upregulated in obese asthmatics compared with non-obese asthmatics, which have been previously reported as obesity-associated marker genes [32,33,34,35,36,37,38].

Most importantly, *IFN-related signaling pathways* were overrepresented in obese asthmatics, compared with both healthy controls and non-obese asthmatics and induced by various IFN-stimulated genes (ISGs) (Figure 3B). Specifically, the involvement of the *interferon-gamma signaling pathway* may indicate that CD4^+^ T cells in obese asthmatics or at least a bigger proportion of them have been skewed in a Th1 polarization manner. 

The lipid metabolism-associated hormone leptin is one principal factor that is able to drive CD4^+^ T cells to produce IFN proteins and might thus, among others, be responsible for this finding [39,40]. This notion is further supported by the observation of the negative correlation between the *IFN signaling* genes expression and FeNO, an established marker of type-2 inflammation in asthma (Figure 4A) [41].

The topological interconnection between *IFN signaling* and *viral infection pathways* (e.g., *influenza factor interaction with host*) in obese asthmatics could define a complex mechanism underlying this asthma phenotype (Figure 2D). Predominant upregulation of *Toll-like receptor pathway regulation* genes such as Toll-like receptor 1 (*TLR-1), TLR-2, TLR-3, TLR-4, TLR-6, and TLR-8* in obese asthmatics compared with healthy controls could be an additional proof of the potential role of viral infections in the low type-2 obesity asthma phenotype (Figure 3B) [42,43]. Tang et al. demonstrated that obesity could foster respiratory tract infections and subsequently contribute to asthma exacerbations in adults [44,45]. Similar findings of the association between obesity and viral infection were reported by Maccioni et al. [46]. Notably, this association was a high spot during the current COVID-19 pandemic because various studies have implied that obesity can worsen the outcomes of the SARS-CoV-2 viral infection [47,48].

Altered regulation of a variety of *GPCR ligand binding* associated genes has been observed in both groups of low type-2 asthmatics compared with healthy controls (Figure 3A) [49,50]. Approximately 34% of currently marketed drugs directly or indirectly target G-protein-coupled receptors [51,52]. The discrete robust correlation between *GPCR ligand binding* enriched genes and most of the lung function parameters in obese asthmatics indicates a key role of the *GPCR signaling pathway* specifically in this asthma phenotype (Figure 4B).

Additional regulation in both asthma groups was observed in genes associated with the *gap junction pathway*, representing another mechanism of cell–cell communication, which plays also a key role in T cell activation (Figure 3A) [53,54,55,56,57]. Shutting down such conventional cellular communication paths could contribute to the persistent inflammatory response in the low type-2 asthma phenotype [57,58]. Of interest, in a mouse model of allergic airway inflammation mimicking asthma, carbenoxolone, a gap junction uncoupling agent, was found to reduce infiltration of inflammatory cells and interleukin production, thereby decreasing lung inflammation [59].

To the best of our knowledge, it is the first study investigating gene expression in obesity-associated asthma on the level of isolated CD4^+^ T cells. Although, the study may be limited by the rather small sample size, it is compensated for by the careful selection of patients representing the phenotype of interest out of a larger cohort. The focus was on a single blood cell population and the immediate isolation of these cells of interest directly after blood drawing and finally deep transcriptome sequencing, resulting in meaningful, solid data and robust, unbiased results.

In conclusion, deciphering the underlying molecular mechanism and thus deeper endo/phenotyping of the unique low type-2 obesity-associated asthma phenotype is a step ahead in the development of modern personalized medicine approaches. Stratified therapy strategies targeting the IFN signaling pathway or some of its central genes, such as the eukaryotic translation initiation factor 2 alpha kinase 2 (*EIF2AK2*), could be a specific treatment option for low type-2 obese asthmatics, whereas conventional or novel anti-viral treatments could be another feasibility. Moreover, certain lung function parameters including FVC, VC max, IC, and FEV1 can be utilized as positive indicators of therapy response in low type-2 obese asthmatics.

## 4. Material and Methods

### 4.1. Study Population and Clinical Assessments

One hundred forty-four asthmatic adults were recruited into the study by the Department of Medicine, Pulmonary and Critical Care Medicine, University Medical Centre Giessen and Marburg. All patients were physician-diagnosed with asthma according to recent clinical guideline diagnostics [21]. Within this cohort, we identified patient sub-groups based on their body mass index (BMI), blood eosinophil counts, and total serum immunoglobulin E (IgE) levels. To selectively focus on low type-2 asthmatics, only patients with low blood eosinophil counts (<300 eos/µL), low fractional exhaled nitric oxide (FeNO; <25 ppb), and low serum total IgE levels (<300 kU/L) were included in this study. Out of those, 10 patients representing the >75% quartile in BMI were selected as obese asthmatics and compared with 10 patients with normal body weight. Detailed patients’ characteristics are shown in Table 1. For comparison, 10 healthy controls were recruited to the Comprehensive Biobank Marburg at the Medical Faculty of the Philipps University of Marburg. Laboratory and clinical parameters including C-reactive protein (CRP), blood cell counts, serum IgE levels, and lung function parameters were measured as part of routine clinical assessments. The study was approved by the local ethics committee (approval No. 155/08/; 202/12), and all participants gave written informed consent.

### 4.2. Blood CD4^+^ T Cells Isolation

For the study purpose, 9 mL of peripheral blood was drawn from all subjects into S-Monovette K3 EDTA collection tubes (Sarstedt, Nümbrecht, Germany). Within 2 h following blood drawing, CD4^+^ T cells were isolated using the StraightFrom^®^ Whole Blood CD4 MicroBeads, human kit (Miltenyi Biotec, Bergisch Gladbach, Germany), according to the manufacturer’s protocol. Quality control was performed by flow cytometry analysis and revealed >95% CD4^+^ T cell purity. Isolated CD4^+^ T cells were lysed in RLT buffer (Qiagen, Hilden, Germany) supplemented with 1% 2-mercaptoethanol (Carl Roth, Karlsruhe, Germany) and preserved at −80 °C until further use.

### 4.3. RNA Extraction and Sequencing

Total RNA was extracted using the miRNeasy Mini Kit (Qiagen) according to the manufacturer’s protocol. RNA quality and quantity were assessed by Agilent Bioanalyzer 2100 (Agilent Technologies, Santa Clara, CA, USA) and Qubit 3 (Thermo Fisher Scientific, Foster City, CA, USA) analyses, respectively. RNA sequencing was performed using the BGISEQ-500 platform (BGI, Shenzhen, China). Briefly, mRNAs molecules were purified from total RNA using oligo(dT)-coupled magnetic beads and fragmented into small pieces using fragmentation reagent. Afterwards, first-strand cDNA was generated using random hexamer-primed reverse transcription, followed by a second-strand cDNA synthesis. The synthesized cDNA was subjected to end-repair and was then 3′ adenylated. Adapters were ligated to the ends of these 3′ adenylated cDNA fragments, which were then amplified using adapter-specific primers. Resulting PCR products were purified with Ampure XP Beads (Beckmann Coulter, Brea, CA, USA) and dissolved in elution buffer. Library quality was validated on an Agilent 2100 Bioanalyzer, and 50 base-pair, paired-end sequencing was performed.

### 4.4. Bioinformatics Analysis

Sequenced reads were mapped with Salmon v1.3.0 in quantification mode against the human transcriptome of cDNA and ncRNA sequences (GRCh38) with the respective genome background as decoys [60]. Resulting read count estimates were rounded to integers, and differential gene expression analysis was performed by DEseq2 [61]. Differential expression analysis was performed with standard parameters, using either asthma status or asthma status + weight as the experimental design. Genes with low counts (<10) were filtered out. For sample–sample comparison and data visualization, cell count data were transformed via Variance stabilizing transformation (vst). iDEP v.0.91 was used to create the correlation matrix of the matched samples as a default option [62]. Heatmaps were created based on Z-score values of gene expression. Functional pathway and gene ontology analyses were conducted by Enrichr Bioplanet and GO biological Process [63,64,65]. Pathway network analyses were built with the R package (ggraph v2.0.5), and gene network analyses were performed by STRING PPI v.11.0 using default settings [66]. Gene networks were further processed by Cytoscape v.23 add-ins MCODE and Clustermaker. Correlation plots of clinical and molecular parameters were generated by the R package (Corrplot v.0.84), implemented with default Pearson correlation.

### 4.5. Statistical Analysis

Unpaired *t*-tests applying a significance threshold of *p* < 0.05 were performed for comparisons of numerical data using the GraphPad Prism 7 software package (GraphPad Software Inc., San Diego, CA, USA).

## Figures and Tables

**Table 1:** General patient characteristics of the two low type-2 asthma patient groups.

	Obese Asthmatics	Non-Obese Asthmatics	*p* Value
N	10	10	1.00
Age (years)	46.30 ± 10.75	39.30 ± 13.94	0.23
Male (%)	30	10	0.29
BMI (kg/m^2^)	36.67 ± 6.90	23.88 ± 2.73	<0.0001
FeNO (ppb)	15.72 ± 7.93	16.78 ± 11.64	0.81
FEV1 (L)	2.89 ± 0.80	2.73 ± 1.10	0.58
FVC (L)	3.79 ± 1.00	3.50 ± 1.00	0.52
VC max (L)	3.96 ± 1.02	3.58 ± 1.00	0.42
PEF (L/s)	6.68 ± 1.54	5.96 ± 2.04	0.38
FEV1/FVC (%)	71.32 ± 4.90	74.09 ± 12.27	0.52
IC (L)	3.36 ± 0.91	2.50 ± 0.67	0.03
CRP (mg/L)	6.80 ± 10.26	1.78 ± 2.06	0.15
Age of onset (years)	37.00 ± 15.63	28.10 ± 14.11	0.20

Data are expressed as mean ± SD. BMI = body mass index; FeNO = fractional exhaled nitric oxide; FEV1 = forced expiratory volume in the first second; FVC = forced vital capacity; VC max = maximum vital capacity; PEF = peak expiratory flow; IC = inspiratory capacity, CRP = C-reactive protein.

**Table 2:** Selected type-2-related genes and their corresponding fold change (FC) and false discovery rate (FDR) values in both comparisons, obese asthmatics vs. Ctrl (healthy controls) and non-obese asthmatics vs. Ctrl.

	Obese Asthmatics vs. Ctrl		Non-Obese Asthmatics vs. Ctrl	
Th2 genes	Log2(FC)	FDR	Log2(FC)	FDR
*GATA3*	−0.489	0.001	−0.446	0.004
*TNFSF11*	−0.026	0.981	0.316	0.753
*IL17RB*	−0.062	0.909	0.083	0.909
*AKAP12*	1.031	0.368	0.916	0.511
*HPGDS*	0.033	0.981	0.331	0.812
*LRRC32*	−0.591	0.222	−0.616	0.258
*PTGDR2*	0.160	0.726	0.598	0.114
*BACE2*	0.048	0.962	0.306	0.768
*NRIP3*	−0.124	0.824	0.058	0.943
*CHDH*	−0.396	0.442	0.106	0.902
*NCS1*	−0.593	0.750	0.293	0.909
*PKP2*	0.250	0.619	0.723	0.094
*LIMA1*	0.088	0.631	0.126	0.533

## Data Availability

The data presented in this study and underlying raw data are available on reasonable request from the corresponding author.

## Supplementary Materials

The following are available online at https://www.mdpi.com/article/10.3390/ijms221810144/s1.
