# Supplementary material for: Differential Regulation of Interferon Signaling Pathways in CD4+ T Cells of the Low Type-2 Obesity-Associated Asthma Phenotype
Source: Int J Mol Sci. 2021 Sep 20;22(18):10144. doi: 10.3390/ijms221810144 (PMC8469911; doi:10.3390/ijms221810144)
Supplement: Supplementary file 1 [file ijms-22-10144-s001.zip › ijms-1383810-supplementary.pdf]

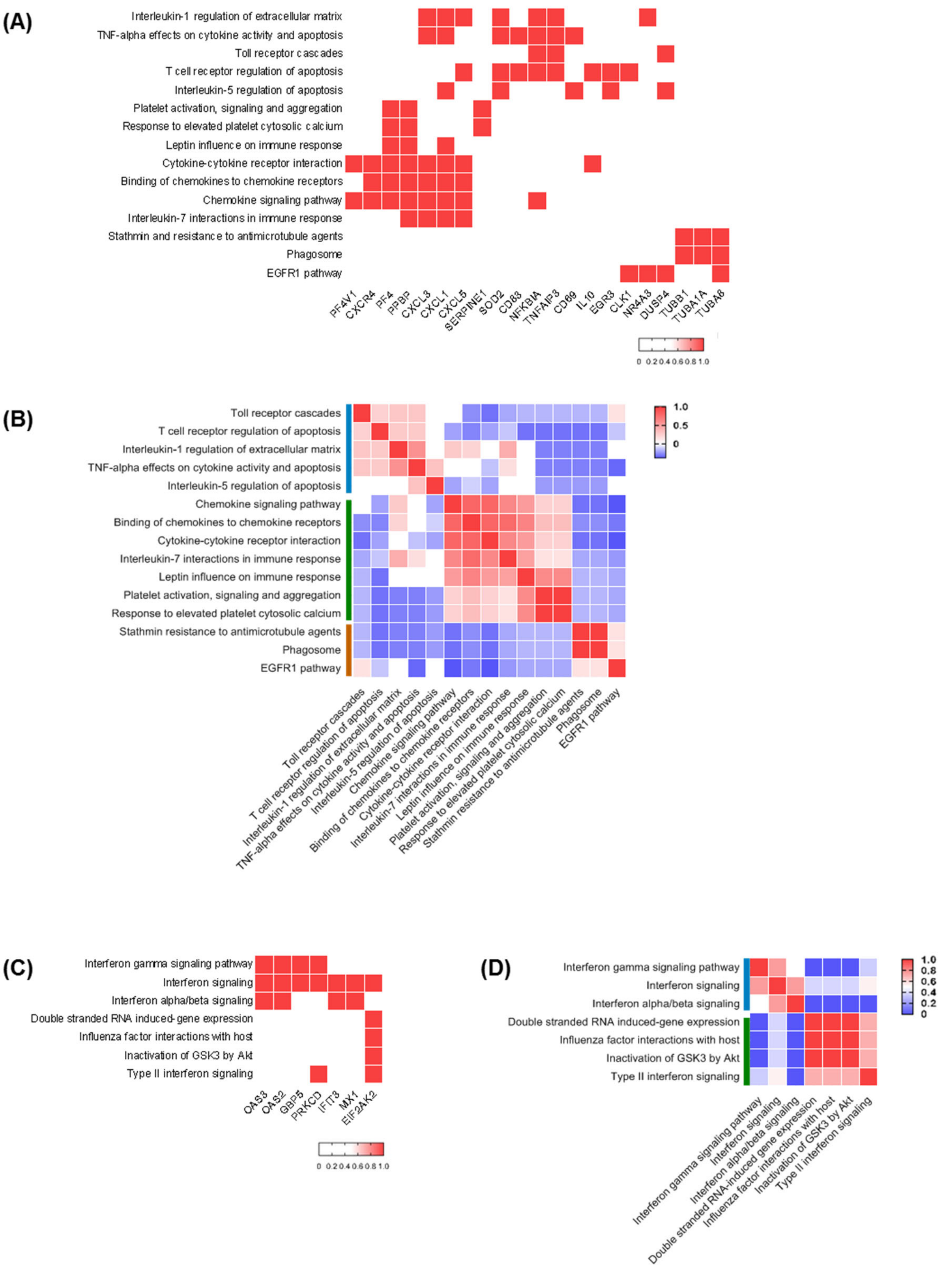
(A)
Interleukin-1 regulation of extracellular matrix
TNF-alpha effects on cytokine activity and apoptosis
Toll receptor cascades
T cell receptor regulation of apoptosis
Interleukin-5 regulation of apoptosis
Platelet activation, signaling and aggregation
Response to elevated platelet cytosolic calcium
Leptin influence on immune response
Cytokine-cytokine receptor interaction
Binding of chemokines to chemokine receptors
Chemokine signaling pathway
Interleukin-7 interactions in immune response
Stathmin and resistance to antimicrotubule agents
Phagosome
EGFR1 pathway
PF4V1
CXCR4
PF4
PPBP
CXCL3
CXCL1
CXCL5
SERPINE1
SOD2
CD83
NFKBIA
TNFAIP3
CD69
IL10
EGR3
CLK1
NR4A3
DUSP4
TUBB1
TUBA1A
TUBA8
0 0.2 0.4 0.6 0.8 1.0
(B)
Toll receptor cascades
T cell receptor regulation of apoptosis
Interleukin-1 regulation of extracellular matrix
TNF-alpha effects on cytokine activity and apoptosis
Interleukin-5 regulation of apoptosis
Chemokine signaling pathway
Binding of chemokines to chemokine receptors
Cytokine-cytokine receptor interaction
Interleukin-7 interactions in immune response
Leptin influence on immune response
Platelet activation, signaling and aggregation
Response to elevated platelet cytosolic calcium
Stathmin resistance to antimicrotubule agents
Phagosome
EGFR1 pathway
1.0
0.5
0
(C)
Interferon gamma signaling pathway
Interferon signaling
Interferon alpha/beta signaling
Double stranded RNA induced-gene expression
Influenza factor interactions with host
Inactivation of GSK3 by Akt
Type II interferon signaling
OAS3
OAS2
GBP5
PRKCD
IFIT3
MX1
EIF2AK2
0 0.2 0.4 0.6 0.8 1.0
(D)
Interferon gamma signaling pathway
Interferon signaling
Interferon alpha/beta signaling
Double stranded RNA induced-gene expression
Influenza factor interactions with host
Inactivation of GSK3 by Akt
Type II interferon signaling
1.0
0.8
0.6
0.4
0.2
0

Figure S1. (A) Mutually shared genes between the top 15 significant biological pathways of obese asthmatics vs. Ctrl (healthy controls) and non-obese asthmatics vs. Ctrl comparisons, and their (B) correlation matrix. (C) Mutual genes between the significant biological pathways of obese asthmatics vs. Ctrl and obese asthmatics vs. non-obese asthmatics comparisons, and their (D) correlation matrix.

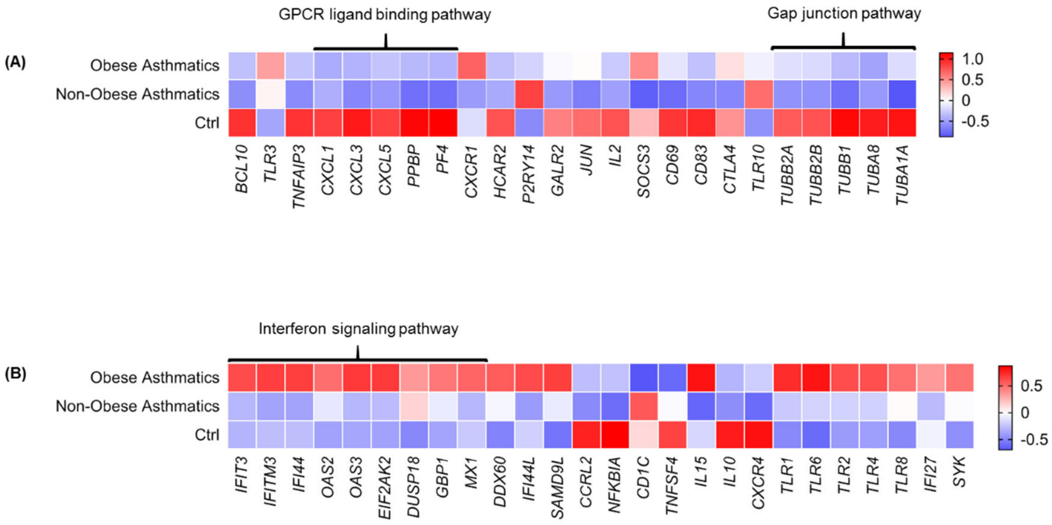

Figure S2. Heatmaps exhibiting the differential gene expression of the gene network clusters associated with three candidate pathways in (A) low type-2 asthma and (B) low type-2 obesity-associated asthma.
